# Seed position in spikelet as a contributing factor to the success of the winter annual invasive grass *Aegilops tauschii*

**DOI:** 10.3389/fpls.2022.916451

**Published:** 2022-08-01

**Authors:** AiBo Wang, Carol C. Baskin, Jerry M. Baskin, Jianqing Ding

**Affiliations:** ^1^State Key Laboratory of Crop Stress Adaptation and Improvement, School of Life Sciences, Henan University, Kaifeng, China; ^2^Department of Biology, University of Kentucky, Lexington, KY, United States; ^3^Department of Plant and Soil Sciences, University of Kentucky, Lexington, KY, United States

**Keywords:** annual grass, invasive species, Poaceae, seed germination, seed position-dependent, effects

## Abstract

Seed position – dependent effects on seed dormancy/germination are well documented at the inflorescence/infructescence level, but less is known about seeds at different positions within a dispersal unit. For the invasive winter annual grass *Aegilops tauschii,* we quantified morphology, mass and dormancy/germination of seeds from basal (1), middle (2), and distal (3) positions in two spikelet types (Left and Right). We also investigated seedling emergence, survival, plant size and seed production of plants from seeds in different spikelet positions of two spikelet types under different soil nutrient and water conditions. We found that these seed, seedling and plant traits performed as mirror images between the Left and Right spikelet types. The middle seed was significantly the longest and had the maximum mass, while the basal seed was the shortest and had medium mass. Middle seeds had the highest increase in mass during imbibition and the highest germination percentages and rates, while basal seeds had the lowest. Seedling emergence and survival, plant size and seed production for each position of seeds were highest in the added fertilizer combined with regular watering treatment and lowest in the no fertilizer combined with natural moisture, while height of plants derived from the middle and the distal seeds was significantly higher than that of plants derived from the basal seeds under all soil nutrient and water conditions. Seedling survival, number of tillers per plant and seed production per plant from the middle and distal seeds were significantly lower than those from basal seeds under all soil nutrient and water treatments. The considerable variation in seedling emergence and survival, plant size and seed production between seeds in different positions in the spikelet results in much flexibility in all stages of the life cycle, thereby likely contributing to the invasiveness of *A. tauschii*.

## Introduction

Variation in seed morphology and dormancy/germination at the level of the inflorescence is well documented, with a general trend for seeds from flowers at the distal positions to be smaller than those from flowers at the basal positions (Winn 1991; Ashman 1992; Wolfe, 1992; Obeso, 1993; Moravcová et al., 2005; Hughes and Simons, 2014). However, less is known about seed variation due to position within a dispersal unit (but see Datta et al., 1970; Wang et al., 2010; Volis, 2014). Variation in plant regeneration from seeds likely favors the spread of introduced alien plant species into new regions and could potentially contribute to their naturalization success (Annamária et al., 2019). However, only relatively few case studies of variation in regeneration from seeds of invasive plant species have been reported (Moravcová et al., 2005; Monty et al., 2016; Qu et al., 2020), and the ecological consequences on invasion success are not well understood.

*Aegilops tauschii* Coss. (syn. *Aegilops squarrosa* L.; Tausch’s goatgrass, Poaceae) is a winter annual weed native to eastern Europe and western Asia (Wang et al., 2021). Wild populations of *A. tauschii* are widely distributed in arid and semi-arid habitats in central Eurasia, occurring from Turkey to western China (Zhou et al., 2021). *A. tauschii* was first recorded in China in 1955 (Yu and Li, 2018), but now it has invaded the key winter wheat growing provinces of Shandong, Shanxi, Shaanxi, Henan and Hebei (Wang, Yuan et al., 2018; Gao et al., 2019; Wang, Tian et al., 2021) and has become one of the most difficult weeds to control in wheat fields (Yu and Li, 2018; Wang et al., 2019). Since *A. tauschii* is one of the wild relatives of wheat, its genome has been widely studied (Huang et al., 2002; Mizuno et al., 2010; Wang et al., 2013; Zhao et al., 2017), and many studies have focused on introduction and utilization of its genetic resources in wheat breeding and improvement (Cox et al., 2017; Kishii, 2019; Zhou et al., 2021). Also, since it is an invasive annual species that propagates only by seeds, seed germination and seedling growth experiments have been carried out by grouping all seeds together (Wang et al., 2018, 2020; Gao et al., 2019), but little or no attention has been given to the position in the spikelet (dispersal unit) where seeds are produced.

**Figure 1:**
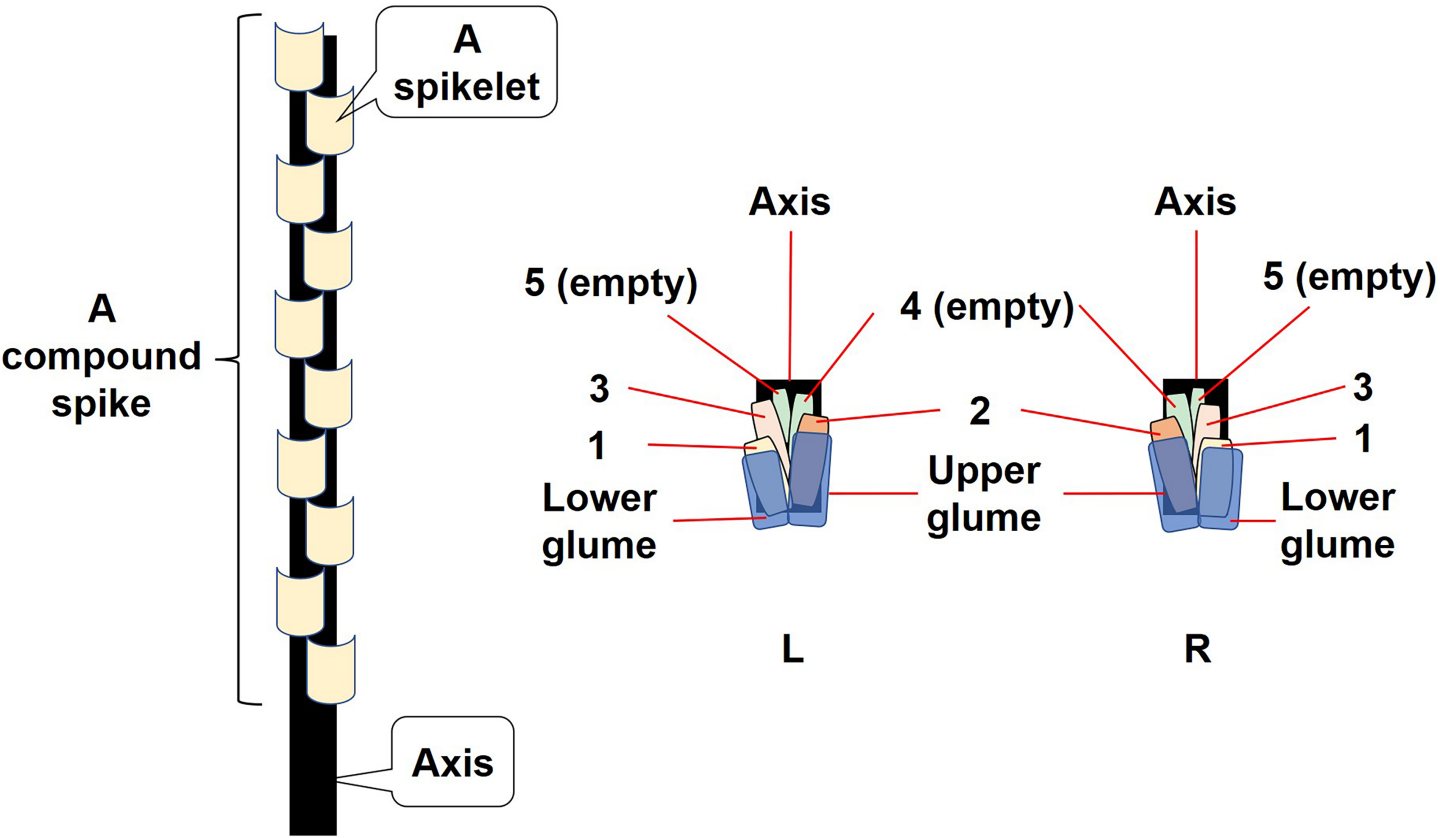
Compound spike and spikelets of *Aegilops tauschii*. The diagram shows the gradation of spikelets in the compound spike. L represents a spikelet with the developmental direction of the basal seed to the left; R represents a spikelet with the developmental direction of the basal seed to the right. Both types of spikelets are dispersed with a small piece of the broken axis attached to them. Numbers 1–5 refer to seeds from the most basal position to the most distal position in a spikelet. The seed in positions 1, 2 and 3 is filled, and these seeds were placed in study groups 1, 2 and 3, respectively. Seeds in position 4 and 5 are not filled.

An individual plant of *A. tauschii* can produce several to dozens of tillers, and each tiller has one compound spike that contains several, even more than 10, spikelets. The spikelets are produced one above the other along the axis of the spike, and the spikelets can be divided into two types (Left and Right, hereafter L and R, respectively; Figure 1) due to the developmental direction of the basal seed. The order of the two types of spikelets on the axis varies. When seeds mature, the axis breaks off node by node, and a spikelet is dispersed with each piece of the axis. Number of florets in a spikelet varies from 1 to 5, and florets in a spikelet are numbered from 1 (the most basal) to 5 (the most distal; Figure 1). The one or two most distal florets in a spikelet do not produce seeds, and approximately 85% of the spikelets contained three filled seeds (A. B. Wang, pers. observ.).

Invasive species generally are more successful under resource-rich than under resource-poor conditions (e.g., Huenneke et al., 1990; Suding et al., 2004; Going et al., 2009; Vallano et al., 2012), and the addition of water increases establishment and survival of some invasive species such as *Holcus lanatus* (Thomson et al., 2006) and *Aegilops triuncialis* (Eskelinen and Harrison, 2014). Since *A. tauschii* has become one of the most difficult weeds to control in wheat fields in China where fertilizer and water (irrigation) are added, we wanted to know the effects of added resources on the growth of *A. tauschii*. Although low levels of both light and soil water can cause decreases in total dry mass per plant of *A. tauschii* seedlings (Wang et al., 2019), seedling/plant performance from seeds in different positions within a spikelet under different nutrient and water conditions is not known.

We hypothesized that: (1) there is variation in morphology and dormancy/germination of seeds in different positions in the spikelets of *A. tauschii*, (2) seeds in different positions give rise to plants that vary in growth, survival and seed production (fitness) in different soil nutrient and water conditions and (3) variation in seeds in different positions results in life history differences that increase adaptiveness of this invasive winter annual.

## Materials and methods

### Study site and seed collection

The study site was an experimental field with sandy soil, which typical of the region where wheat is grown, located on the campus of Henan University, Kaifeng, Henan Province, China (E: 114.23, N: 34.52, altitude 73 m). All spikelets were collected from about 3,000 individual plants of *A. tauschii* growing at the study site on June 8–12, 2020, at which time seeds were fully matured. The two types of spikelets and their seeds were used in this study, and seeds in positions 1 (basal), 2 (middle), and 3 (distal) of a spikelet are referred to as groups 1, 2, and 3, respectively.

### Seed morphology and mass

We determined dimensions and mass of caryopses (hereafter seeds) in each of the three groups of seeds and associated structures in both types of spikelets. The seeds, axes, lower and upper glumes and lemmas, and paleae were removed from 50 spikelets of each type and their length and width measured using vernier calipers. Length of awns also was measured. Seeds and the associated structures were separated from four replicates of 100 spikelets of both L and R types (Figure 1) and weighed using an analytical balance (0.0001 g).

### Water uptake (imbibition)

To measure water uptake (imbibition), fresh seeds (with and without lemmas and paleae) and structures separated from four replicates of 100 spikelets of both types 2 weeks after collection were placed on Whatman No. 1 filter paper moistened with distilled water in 12 cm diameter Petri dishes at room conditions. At time 0 and at 1-h intervals until final constant mass was reached, structures and seeds were removed from the dishes, blotted dry with filter paper, weighed with an analytical balance to the nearest 0.0001 g and returned to the Petri dishes. Percentage increase in mass (water uptake) was calculated by dividing the increase in mass by the original mass.

### Germination of fresh seeds

For both kinds of spikelets, four replicates of 25 fresh seeds (2 weeks after collection) of each group separated from spikelets (separated seeds without lemmas and paleae) and 25 intact spikelets with the seeds inside were placed on two layers of Whatman No. 1 filter paper moistened with distilled water in 12-cm diameter Petri dishes. Seeds were incubated at daily temperature regimes of 15/6, 22/12, 28/18, and 32/24°C in light (12-h daily photoperiod, c. 100 μmol m^−2^ s^−1^, 400–700 nm, cool white fluorescent light) or in constant darkness (Petri dishes placed in black bags) for 28 days. A seed was considered to be germinated when the coleorhiza (with a shoot-born root inside it) had emerged (see Tillich, 2007). The four temperature regimes approximate mean daily maximum and minimum monthly air temperatures in the city of Kaifeng during the growing season: late February, March, and November, 15/6°C; April and October, 22/12°C; May and September, 28/18°C; June, July, and August, 32/24°C.^1^

Germination in light of seeds separated from spikelets was examined daily for 28 days, and any seeds that had germinated were removed from the dishes; distilled water was added when needed. It was difficult to check germination of seeds in the intact spikelets each day; thus, germination of seeds in intact spikelets incubated in light as well as those incubated in darkness was checked only after 28 days. The rate (speed) of germination in light of separated seeds was estimated using a modified Timson index of germination velocity: germination index = ∑*G*/*t*, where *G* is seed germination percentage at 1-day intervals, and *t* is the germination period (Timson, 1965). A high value indicates rapid germination, with the highest value obtainable being 100, when 100% of the seeds germinated on the first day (i.e., 2800/28).

### Effect of afterripening on dormancy-break/germination

Germination of fresh separated seeds and seeds within intact spikelets in light and in darkness across all temperature regimes was 7%–80% and 0%–66.8%, respectively, while seed viability tests using the tetrazolium test gave a positive test in 95% of the non-germinated seeds, indicating that a portion of the seeds was dormant. Dormancy-break by afterripening is one of the characteristics of seeds with nondeep physiological dormancy (PD; Baskin and Baskin, 2014). Thus, we determined if seeds come out of dormancy (after-ripen) during dry storage. Seeds were stored for 0 (control) and 12 months in a closed cotton bag under room conditions (15°C–25°C, 20%–35% relative humidity), beginning June 26, 2020. After dry storage, four replicates of 25 separated seeds of each group and 25 intact spikelets with seeds inside were incubated in Petri dishes on two layers of filter paper moistened with distilled water at each of the four temperature regimes in light and in constant darkness for 28 days.

### Seedling emergence and survival, plant size and seed production

On October 18, 2020, 80 separated seeds of each of the three seed groups from spikelet types L and R were sown in 16 1.8-m long × 1.5-m wide plots in sandy soil in the experimental field. In the nonfertilized soil, total nitrogen content was analyzed using an elemental analyzer (Perkin Elmer 2400, United States), total phosphorus content was determined by the molybdenum blue colorimetry method after digestion with perchloric and sulfuric acid (John, 1970) and the rapidly available potassium was extracted with ammonium acetate and measured by atomic absorption spectrometry (Knudsen et al., 1982). The content of nitrogen, phosphorus and potassium was 1.2740, 0.5244 and 0.0690 g/kg, respectively, which represents the mean level of soil nutrients in the local wheat farmland. Before sowing, the soil was ploughed to a depth of 30 cm, and fertilizer (75 g/m^2^ of 10:10:10 N, P and K fertilizer, which is the same as that used in the local cultivation of wheat) was added to eight plots for each seed group, while the other eight plots of each seed group were not fertilized (control). In each soil nutrient condition, four plots for each seed group received only natural precipitation, while the other four were watered to field capacity weekly, except from December to mid-February, when seeds received moisture only from snow and rain; evaporation was very low. Thus, there were four soil conditions: added fertilizer and watered regularly (high nutrient and high water, H–H); added fertilizer and no water (H–L); no fertilizer and watered regularly (L–H); and no fertilizer and no water (L–L). Emergence of seedlings was recorded weekly until June 1, 2020, by which time plants had flowered and set seeds. Then, height of plants, number of tillers per surviving plant, spikelets per tiller and seeds per spikelet were recorded. Number of seeds per plant and number of seeds from all surviving plants were calculated. Temperature, rainfall and evaporation data were obtained from the China meteorological administration (see Footnote 1).

### Statistical analysis

Generalized linear model (GLM) was used to test for significance of the (1) main effects (spikelet type and seed position) and their interaction on size and mass of seeds and associated structures; (2) main effects [spikelet type, seed position and treatment (seeds with or without lemmas and paleae)] and their interaction on percentage of increase in mass of seeds during water absorption; (3) main effects (spikelet type, seed position and temperature) and their interaction on germination index of fresh seeds; (4) main effects (spikelet type, seed position, light and temperature) and their interaction on germination percentage of fresh seeds; (5) main effects [seed age (fresh seeds or seeds after 12 months of dry storage), light, temperature, spikelet type and seed position] and their interaction on germination percentages; (6) main effects [seed age (fresh seeds or seeds after 12 months of dry storage), temperature, spikelet type and seed position] and their interaction on germination index of seeds; and (7) main effects (spikelet type, seed position, soil condition and season) and their interaction on seedling emergence, seedling survival, plant size and seed production.

To determine the impacts of seed position on dimensions and mass of seeds and associated structures, final percentage of increase in mass during water absorption, germination index and percentages of fresh seeds and 12 months dry stored seeds, seedling emergence, seedling survival, plant size and seed production, the impact of spikelet type on dimensions and mass of axes, lower glumes and upper glumes, and the differences of dimensions and mass between lower glumes and upper glumes, one-way analysis of variance (ANOVA) was applied to determine the differences of these traits among different seeds in different positions of spikelets. All data were analyzed for normality and homogeneity of variance prior to analysis to fulfill requirements for ANOVA. If data were normal and homogeneous, they were subjected to further analysis. If data exhibited abnormal distribution or if variances were not homogeneous, they were log_10_ or square-root transformed before analysis to ensure homogeneity of variance (non-transformed data appear in all tables and figures).

The data were analyzed using the software R, version 3.4.2. Tukey’s HSD test was performed for multiple comparisons to test for significant (*p* < 0.05) differences between individual treatments.

## Results

### Seed morphology and mass

#### Seed dimensions

Seed position significantly affected length of lemmas (*p* < 0.001), length of paleae (*p* < 0.001), width of lemmas (*p* < 0.001), width of paleae (*p* < 0.001), length of awns (*p* < 0.001), length of seeds (*p* < 0.001), width of seeds (*p* < 0.001) and thickness of seeds (*p* = 0.001), while spikelet type (*p* = 0.908, *p* = 0.912, *p* = 0.101, *p* = 0.878, *p* = 0.939, *p* = 0.971, *p* = 0.402, *p* = 0.338), and the interaction between spikelet type and seed position [*p* = 0.976, *p* = 0.460, *p* = 0.217, *p* = 0.936, *p* = 0.220, *p* = 0.499, *p* = 0.338, except width of lemmas (*p* = 0.029)] did not affect these indices significantly.

**Figure 2:**
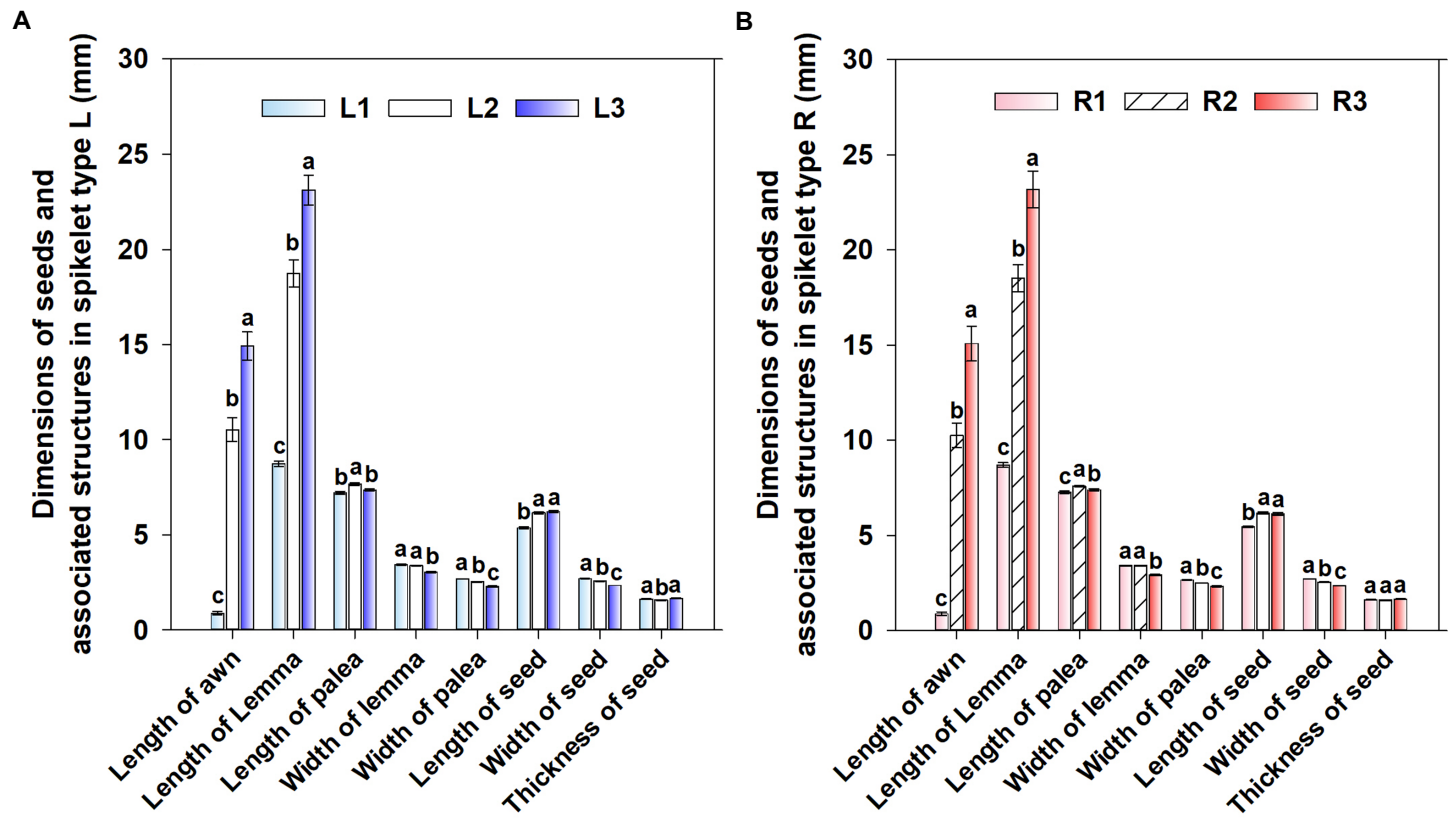
Dimensions (mm, mean ± standard deviation) of seeds and associated structures in the spikelet type L **(A)** and R **(B)** of *Aegilops tauschii* (*n* = 50). L, spikelet type Left; R, spikelet type Right; numbers 1, 2, and 3 following abbreviations indicate different seed groups. Different letters between seed groups for each dimension and each type of spikelet indicate significant differences (Tukey’s HSD, *p* = 0.05).

For both spikelet types, length of awns and lemmas was significantly the highest in group 3 and significantly the lowest in group 1. However, width of paleae and seeds without lemmas and paleae was significantly the highest in group 1 and significantly the lowest in group 3. Length of paleae was significantly higher in group 2, while width of lemmas and length of seeds without lemmas and paleae was significantly lower in group 3 and group 1, respectively. However, in spikelet type L thickness of seeds without associated structures was significantly lower in group 2, while it was not significantly different among the three groups in spikelet type R (Figure 2). Length (mm) (type L: 10.174 ± 0.177, type R: 9.999 ± 0.212; *p* = 0.267) and diameter (mm) (type L: 3.017 ± 0.052, type R: 3.156 ± 0.067; *p* = 0.118) of axes were not significantly different between the two spikelet types (these data are not shown in Figure 2). Spikelet type did not significantly affect the length (*p* = 0.842, *p* = 0.647) or width (*p* = 0.389, *p* = 0.106) of the lower or upper glumes, but the length (mm) (type L: 7.22 ± 0.058, type R: 7.314 ± 0.108) and width (mm) (type L: 3.239 ± 0.045, type R: 3.354 ± 0.057) of the upper glumes were significantly (except for length of glumes in type R) larger than those of the lower glumes (the lengths of lower glumes were 6.959 ± 0.048 and 7.112 ± 0.116 in type L and type R, respectively, the widths of lower glumes were 3.108 ± 0.041and 3.062 ± 0.045 in type L and type R, respectively; these data are not shown in Figure 2).

#### Seed mass

Seed position significantly affected mass of lemmas (*p* < 0.001), paleae (*p* < 0.001), seeds without lemmas and paleae (*p* < 0.001), and seeds with lemmas and paleae (*p* < 0.001), while spikelet type did not (*p* = 0.968, *p* = 0.111, *p* = 0.612, *p* = 0.749). The interaction between seed position and spikelet type only significantly affected mass of lemmas (*p* = 0.002).

**Figure 3:**
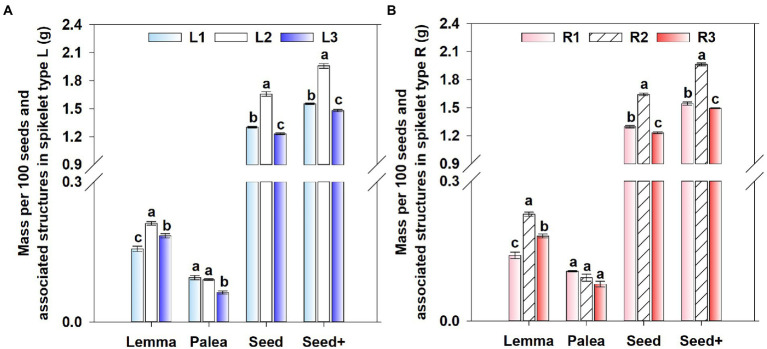
Mass per 100 seeds and associated structures in the spikelet type L **(A)** and R **(B)** of *Aegilops tauschii*. L, spikelet type Left; R, spikelet type Right; Seed, seed without lemma and Palea; Seed+, seed with lemma and palea; numbers 1, 2 and 3 following abbreviations indicate different seed groups. Different lowercase letters between seed groups for each dimension and each type of spikelet indicate significant differences (Tukey’s HSD, *p* = 0.05).

For mass of lemmas, group 2 was significantly > group 3 significantly > group 1, and for mass of paleae, group 1 > group 2 significantly (except for type R) > group 3 (Figure 3). For mass of seeds with and without lemmas and paleae, group 2 was significantly > group 1 significantly > group 3 (Figure 3). For both types of spikelets, mass of the upper glumes was significantly greater than that of the lower glumes, but mass (g) of axes (type L: 1.1636 ± 0.0422, type R: 1.1563 ± 0.0421; *p* = 0.998), lower glumes (type L: 0.9368 ± 0.0251, type R: 0.9083 ± 0.0154; *p* = 0.539), or upper glumes (type L: 0.9496 ± 0.0181, type R: 0.9211 ± 0.0193; *p* = 0.387) were not significantly different between the two spikelet types (these data are not shown in Figure 3).

### Water absorption

Seed position (*p* < 0.001) and treatment (seeds with or without lemmas and paleae; *p* < 0.001) significantly affected final percentage of increase in mass, while spikelet type (*p* = 0.782), the interaction between treatment and spikelet type (*p* = 0.862), the interaction between spikelet type and seed position (*p* = 0.918), the interaction between treatment and seed position (*p* = 0.314) and the interaction between treatment, spikelet type and seed position (*p* = 0.817) did not.

**Figure 4:**
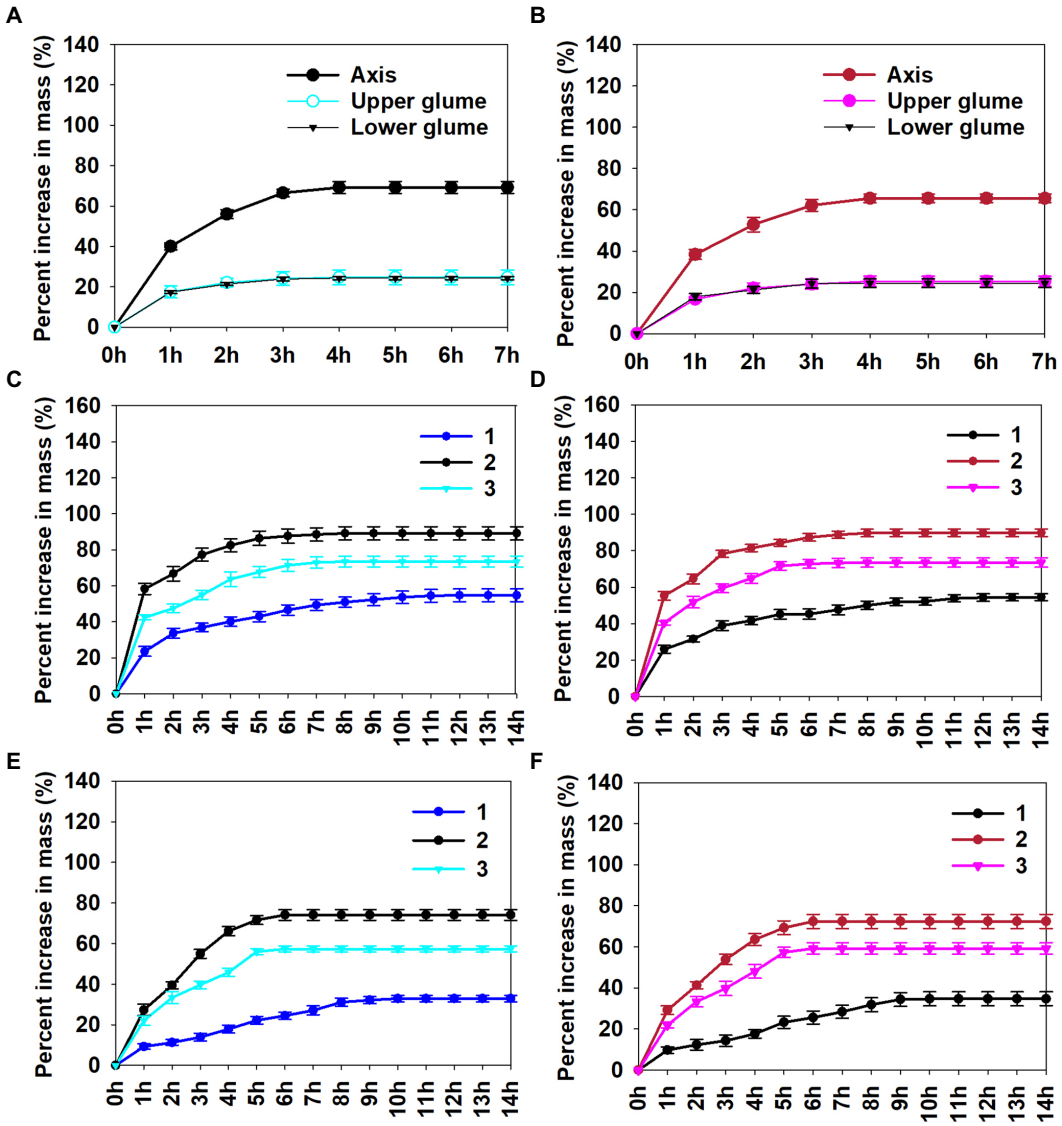
Cumulative percent increase in mass *via* imbibition of water by three associated structures **(A, B)**, seeds with **(C, D)** and without **(E, F)** lemmas and paleae of Left **(A, C, E)** and Right **(B, D, F)** spikelet types in *Aegilops tauschii*. Numbers 1, 2 and 3 following abbreviations indicate different seed groups.

The axes (*p* = 0.422), lower glumes (*p* = 0.902), and upper glumes (*p* = 0.988) did not differ significantly in final percentage of increase in mass between the two spikelet types. Axes, lower glumes and upper glumes all stopped absorbing water within 4 h, while seeds with and without lemmas and paleae were fully imbibed after 6–12 h, depending on seed group (Figure 4). Three groups of seeds with lemmas and paleae had a high capacity for water uptake, and mass increased 54.5%–89.8% within 8–12 h, which differed significantly from that of seeds without lemmas and paleae. Moreover, the percentage increase in mass of water imbibed by seeds with and without lemmas and paleae differed significantly among seed groups, group 2 was the highest, while group 1 was significantly the lowest.

### Germination of fresh seeds

**Figure 5:**
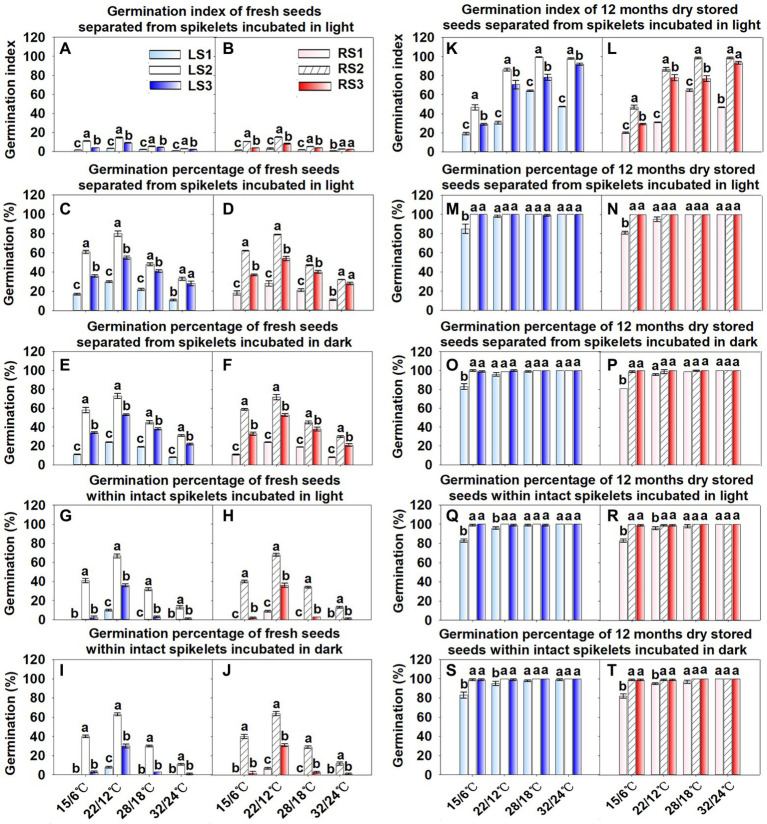
Germination percentages and germination index of fresh (o months storage; **A–J**), and of 12 months dry—stored **(K–T)** seeds of the three seed groups. LS, seeds in spikelet type Left; RS, seeds in spikelet type Right; numbers 1, 2 and 3 following abbreviations indicate different seed groups; seed groups within a temperature of a storage category with different letters are significantly different (Tukey’s HSD, *p* = 0.05).

Temperature (*p* < 0.001), seed position in spikelets (*p* < 0.001) and treatment (seeds separated or in the intact spikelet; *p* < 0.001) significantly affected germination percentages of seeds, while spikelet type (*p* = 0.964) and light (*p* = 0.267) did not (Figures 5A–J). Temperature (*p* < 0.001) and seed position in spikelet (*p* < 0.001) had significant effects on the germination index, while spikelet type (*p* = 0.977) did not (Figures 5A–J). The interactions between temperature and seed position (*p* < 0.001), temperature and treatment (*p* < 0.001), temperature and light (*p* = 0.020), seed position and treatment (*p* < 0.001), treatment and light (*p* < 0.001), and temperature, seed position, and treatment (*p* < 0.001) significantly affected germination percentages of fresh seeds. The interactions between temperature and position (*p* < 0.001) significantly affected the germination index of fresh seeds.

All three groups of seeds from both types of spikelets germinated best at 22/12°C and worst at 32/24°C. Group 2 seed germination percentages were higher at 15/6°C than at other temperatures, while germination percentages and germination index were similar at 15/6°C and 28/18°C for groups 1 and 3 seeds (Figures 5A–J). Compared to seeds separated, germination percentages of fresh groups 1 and 3 seeds not separated from spikelets decreased sharply at all temperatures, while those of group 2 decreased gently (Figures 5C–J). Overall, germination percentages and germination index of group 2 seeds were significantly the highest, and those of group 1 seeds were significantly the lowest (Figures 5A–J). That is, group 2 had the highest proportion of nondormant seeds and group 1 the highest proportion of dormant seeds.

### Effect of afterripening on dormancy-break/germination

Germination percentages (*p* < 0.001) and germination index (*p* < 0.001) of seeds dry stored for 12 months increased significantly compared to fresh seeds (Figure 5). Temperature (*p* < 0.001) and seed position in spikelets (*p* < 0.001) significantly affected germination percentages, while spikelet type (*p* = 0.903), treatment (seeds separated or in the intact spikelet; *p* = 0.146) and light (*p* = 0.245) did not (Figures 5K–T). Temperature (*p* < 0.001) and seed position in spikelet (*p* < 0.001) had significant effects on the germination index, but spikelet type (*p* = 0.852) did not (Figures 5K–T). The only interaction that significantly affected germination percentages (*p* < 0.001) and germination index (*p* < 0.001) was between temperature and seed position.

Germination percentages of groups 2 and 3 seeds were significantly higher than that of group 1 for separated seeds at 15/6°C (Figures 5M–P) and for seeds in intact spikelets at 15/6°C and 22/12°C (Figures 5Q–T). The germination index was significantly the highest for group 2 and significantly the lowest for group 1 seeds (Figures 5K,L). Group 1 seeds had the lowest and highest germination index at 15/2°C and 28/18°C, respectively, while germination percentages were similar at the four temperature regimes (Figures 5K–T). The germination index increased as temperature increased for groups 2 and 3 seeds (Figures 5K,L). Compared to seeds separated (Figures 5M–P), germination percentage of 12-month dry-stored groups 1, 2, and 3 seeds not separated from the spikelets did not change significantly (Figures 5Q–T).

### Seedling emergence and survival, plant size and seed production

#### Seedling emergence and survival

**Figure 6:**
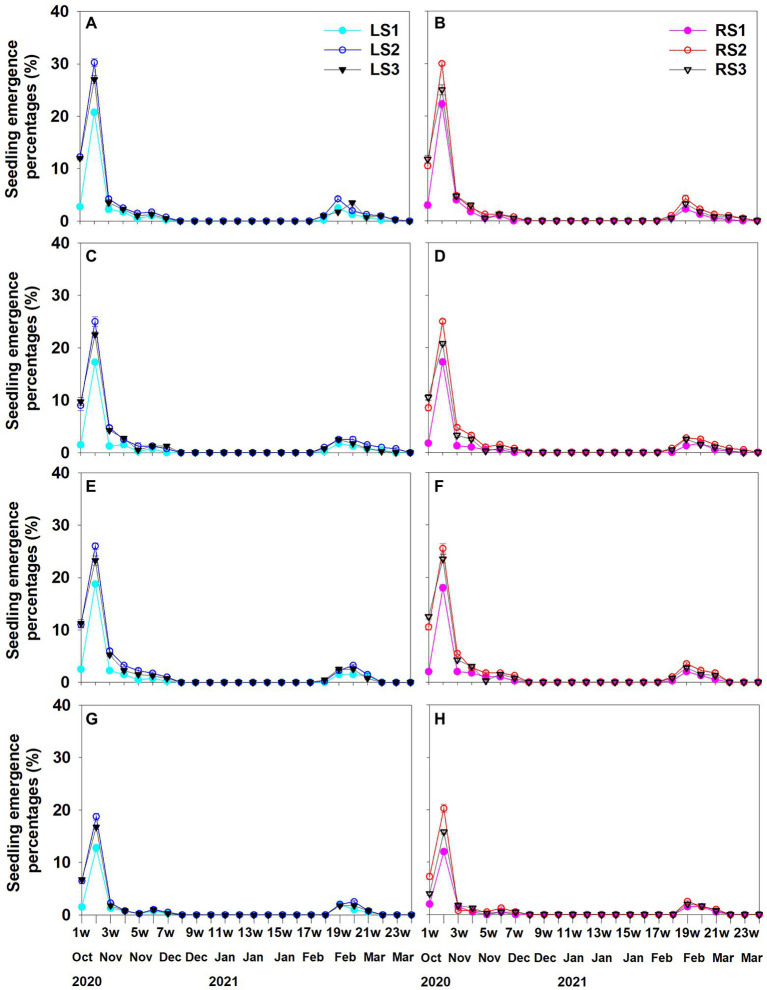
Seedling emergence from seeds separated from Left **(A, C, E, G)** and Right **(B, D, F, H)** spikelet types of *Aegilops tauschii* sown in field plots on October 18, 2020. **(A, B)** seedling emergence under added fertilizer and watered regularly condition; **(C, D)** seedling emergence under added fertilizer and natural moisture condition; **(E, F)** seedling emergence under no fertilizer and watered regularly condition; **(G, H)** seedling emergence under no fertilizer and natural moisture conditions; LS, seedlings derived from seeds in spikelet type Left; RS, seedlings derived from seeds in spikelet type Right. Numbers 1, 2 or 3 following LS and RS indicate different seed groups.

In the four soil conditions, seeds of each group germinated in autumn and in spring, with most seeds germinating in autumn and only a few in spring (Figure 6). The peak period of autumn and spring seedling emergence was in October (week 2 after sowing) and mid-to-late February (weeks 19–20 after sowing), respectively.

Soil condition (*p* = 0.007, *p* < 0.001), seedling emergence season (autumn and spring; *p* < 0.001, *p* < 0.001) and seed position in spikelet (*p* < 0.001, *p* < 0.001) significantly affected percentages of seedling emergence and survival, but there was no significant difference between the two spikelet types (*p* = 0.767, *p* = 0.957). Interactions that significantly affected seedling emergence percentages were between season and soil condition (*p* < 0.001), season and seed position (*p* < 0.001), soil condition and seed position (*p* < 0.001) and season, soil condition and seed position (*p* < 0.001). The interactions between season and seed position (*p* < 0.001) and between soil condition and seed position (*p* = 0.004) significantly affected seedling survival percentages.

**Figure 7:**
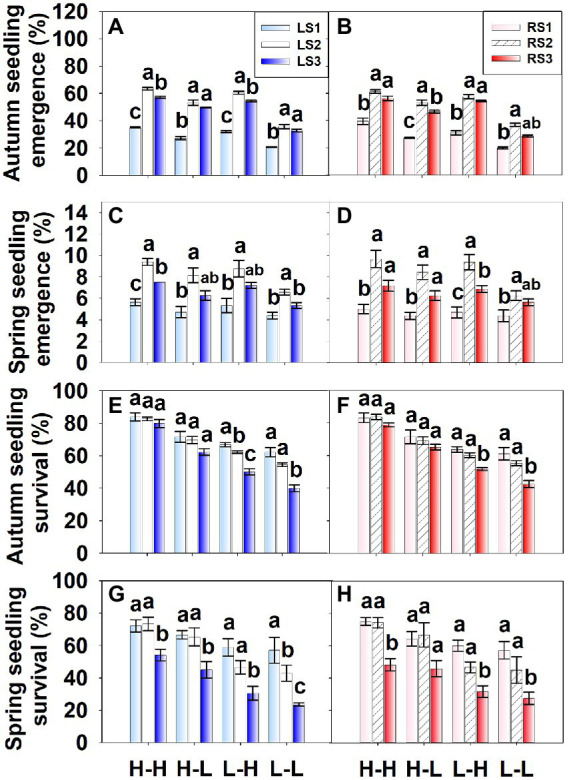
Seedling emergence percentages (from seeds separated from spikelets) and survival percentages of *Aegilops tauschii* in field plots. H-H, added fertilizer, watered regularly; H-L, added fertilizer, natural moisture; L-H, no fertilizer, watered regularly; L-L, no fertilizer, natural moisture; LS, spikelet type Left; RS, spikelet type Right. Numbers 1, 2 or 3 following LS and RS indicate different seed groups. Seed groups within a soil condition for L and R with different letters are significantly different (Tukey’s HSD, *p* = 0.05).

For each seed group, percentages of seedling emergence (*p* < 0.001) and survival (*p* < 0.001) were significantly higher for autumn germination than for spring germination. Seedling emergence percentages were significantly the highest for group 2 seeds and significantly the lowest for group 1 seeds (Figures 7A–D), while seedling survival percentages were significantly the highest for group 1 (except for a few cases) and significantly the lowest for group 3 seeds (Figures 7E–H). For each spikelet type and each season, seedling emergence percentages for each seed group were H-H > L-H > H-L > L-L (Figures 7A–D) and for seedling survival percentages for each seed group were H-H > H-L > L-H > L-L (Figures 7E–H).

#### Plant size and seed production

Season (*p* < 0.001, *p* < 0.001, *p* < 0.001, *p* < 0.001), seed position in spikelet (*p* < 0.001, *p* < 0.001, *p* < 0.001, *p* < 0.001), and soil condition (*p* < 0.001, *p* < 0.001, *p* < 0.001, *p* < 0.001) had significant effects on height of plants, number of tillers per plant, number of spikelets per tiller and number of seeds per spikelet, respectively. However, spikelet type did not significantly affect plant height (*p* = 0.980), number of tillers per plant (*p* = 0.900), number of spikelets per tiller (*p* = 0.841) or number of seeds per spikelet (*p* = 0.777). Among all interactions, only the one between season and soil condition (*p* = 0.001) significantly affected plant height. The interactions between season and soil condition (*p* < 0.001) and between soil condition and seed position (*p* < 0.001) significantly affected the number of tillers per plant, and the interaction between season and seed position (*p* < 0.001) significantly affected the number of seeds per spikelet.

In both spikelet types and under the four soil conditions, all growth indices were significantly (*p* < 0.001) higher for plants derived from seedlings emerging in autumn than in spring. In both spikelet types and for plants from seedlings emerging in both seasons, all growth indices were H-H > H-L > L-H > L-L. In all treatments, height of plants and number of spikelets per tiller were significantly the highest for group 2 and significantly the lowest for group 1 seeds. The number of tillers per plant and number of seeds per spikelet were significantly the highest in group 1 (except in a few cases) and the lowest in group 3 seeds (Tables 1 and 2). The surviving plants from group 1 seeds produced more seeds per plant than those from either group 2 or group 3 seeds. However, seedling emergence percentages and total number of surviving plants from group 2 seeds were higher than those from either group 1 or 3, and all surviving plants from groups 1 and 3 seeds produced fewer seeds than surviving plants from group 2 seeds (Tables 1 and 2).

**Table 1:** Plant size and seed production of *Aegilops tauschii* from autumn germinating seeds.

Autumn emergence	Soil condition	LS1	LS2	LS3	*p-*value	RS1	RS2	RS3	*p*-value
Height	H-H	51.53 ± 2.29	66.13 ± 1.75	62.9 ± 2.21	0.021	54.38 ± 1.23	67.25 ± 1.85	63.9 ± 4.3	**0.077**
	H-L	48.38 ± 1.36	57.9 ± 1.08	54.75 ± 1	0.018	47.53 ± 0.36	59.8 ± 2.35	55.68 ± 0.54	0.015
	L-H	40.78 ± 1.35	50.1 ± 1.92	44.63 ± 1.52	0.030	44.1 ± 0.87	48.03 ± 2.26	44.1 ± 1.36	**0.236**
	L-L	35.3 ± 1.56	44 ± 1.07	39.55 ± 1.33	0.017	33.43 ± 2.52	43.6 ± 1	39.78 ± 0.79	0.023
	*p*-value	0.009	0.004	0.004		0.003	0.007	0.006	
No. of tillers per plant	H-H	53.75 ± 2.53	52.5 ± 1.48	40 ± 1.54	0.024	54.5 ± 2.36	51.25 ± 1.29	38.5 ± 2.51	0.027
	H-L	43.75 ± 2.01	40 ± 2.24	33 ± 1.54	0.033	46.25 ± 3.21	43.25 ± 2.56	33.25 ± 1.14	0.031
	L-H	34.75 ± 1.56	30.75 ± 1.63	24 ± 0.79	0.020	36.25 ± 1.47	30 ± 1.46	24.75 ± 0.74	0.013
	L-L	30.75 ± 1.19	25 ± 1.27	21.75 ± 0.74	0.019	30.5 ± 0.9	24 ± 1.12	22 ± 0.79	0.018
	*p*-value	0.005	0.004	0.005		0.005	0.004	0.005	
No. of spikelets per tiller	H-H	10.73 ± 0.15	11.69 ± 0.14	10.91 ± 0.14	0.000	10.72 ± 0.15	11.59 ± 0.12	10.92 ± 0.13	0.000
	H-L	10.39 ± 0.16	11.22 ± 0.14	10.66 ± 0.15	0.000	10.42 ± 0.17	11.14 ± 0.1	10.64 ± 0.15	0.000
	L-H	10.06 ± 0.16	10.8 ± 0.14	10.28 ± 0.18	0.002	10.19 ± 0.17	10.44 ± 0.15	10.33 ± 0.15	**0.428**
	L-L	10.03 ± 0.18	10.42 ± 0.14	10.23 ± 0.18	**0.228**	10.08 ± 0.16	10.3 ± 0.14	10.19 ± 0.14	**0.673**
	*p*-value	0.003	0.000	0.013		0.033	0.000	0.000	
No. of seeds per spikelet	H-H	3.7 ± 0.08	3.2 ± 0.06	2.8 ± 0.05	0.000	3.69 ± 0.1	3.24 ± 0.04	2.78 ± 0.05	0.000
	H-L	3.62 ± 0.1	3.02 ± 0.06	2.59 ± 0.06	0.000	3.57 ± 0.11	3.06 ± 0.06	2.65 ± 0.06	0.000
	L-H	3.47 ± 0.11	2.98 ± 0.04	2.54 ± 0.06	0.000	3.5 ± 0.11	3 ± 0.04	2.53 ± 0.07	0.000
	L-L	3.32 ± 0.1	2.69 ± 0.06	2.46 ± 0.08	0.000	3.38 ± 0.1	2.71 ± 0.07	2.58 ± 0.06	0.000
	*p-*value	0.016	0.000	0.003		**0.223**	0.000	0.011	
No. of seeds per plant	H-H	2133.929	1963.92	1221.92		2155.846	1924.52	1168.768	
	H-L	1645.516	1355.376	911.1102		1720.472	1474.323	937.517	
	L-H	1213.06	989.658	626.6688		1292.856	939.6	646.8388	
	L-L	1023.963	700.745	547.3562		1039.147	669.912	578.3844	
Total no. of seeds from all plants	H-H	50147.33	82484.64	44600.08		56051.99	78905.3	41491.25	
	H-L	25505.5	39983.59	22549.98		26667.32	43123.96	22734.79	
	L-H	20622.02	29937.15	13630.05		20362.49	26073.9	14553.87	
	L-L	10495.62	10861.55	5747.24		10131.69	10886.07	5639.248	

H-H, added fertilizer, watered regularly; H-L, added fertilizer, natural moisture; L-H, no fertilizer added, watered regularly; L-L, no fertilizer added, natural moisture; LS, spikelet type Left; RS, spikelet type Right; numbers 1, 2 or 3 following LS and RS indicate different seed groups. *p*-values in bold mean differences were not significant.

**Table 2:** Plant size and seed production of *Aegilops tauschii* from spring germinating seeds.

Spring emergence	Soil condition	LS1	LS2	LS3	*p*-value	RS1	RS2	RS3	*p*-value
Height	H-H	40.63 ± 1.4	48.23 ± 0.27	43.93 ± 1.05	0.015	39.08 ± 0.77	49.95 ± 0.86	43.23 ± 1.26	0.012
	H-L	30.35 ± 1.2	41.58 ± 1.21	37.05 ± 0.84	0.010	29.85 ± 1.68	40.2 ± 1.82	36.58 ± 1.4	0.026
	L-H	29.08 ± 0.93	36.43 ± 0.62	33 ± 1.5	0.030	26.88 ± 1.9	38.05 ± 2.11	30.08 ± 0.75	0.050
	L-L	17.9 ± 1.16	27.35 ± 1.81	21.9 ± 0.58	0.017	19.08 ± 1.58	26.13 ± 1.14	22.83 ± 0.31	0.077
	*p*-value	0.005	0.003	0.004		0.007	0.005	0.003	
No. of tillers per plant	H-H	40.5 ± 2.08	37 ± 1.54	27.5 ± 1.64	0.018	40.25 ± 2.07	36.75 ± 2.22	29.5 ± 1.75	**0.309**
	H-L	35.5 ± 1.35	29.25 ± 1.43	23.5 ± 1.68	0.015	34 ± 1.27	27.75 ± 1.19	24 ± 0.94	0.043
	L-H	30.75 ± 1.67	20.5 ± 1.15	17.5 ± 0.56	0.012	31 ± 1.54	22.5 ± 1.09	16.25 ± 1.08	0.02
	L-L	25.25 ± 1.47	19.75 ± 0.74	17.5 ± 0.56	0.014	24.75 ± 1.52	20.5 ± 0.56	18.5 ± 0.83	0.059
	*p*-value	0.010	0.005	0.008		0.010	0.005	0.005	
No. of spikelets per tiller	H-H	10.52 ± 0.12	11.03 ± 0.14	10.7 ± 0.11	0.044	10.53 ± 0.11	10.98 ± 0.13	10.7 ± 0.12	0.0200
	H-L	10.25 ± 0.13	10.77 ± 0.13	10.34 ± 0.13	0.015	10.22 ± 0.15	10.72 ± 0.11	10.33 ± 0.12	0.008
	L-H	10 ± 0.14	10.5 ± 0.11	10.14 ± 0.12	0.007	10.06 ± 0.14	10.48 ± 0.1	10.17 ± 0.13	0.014
	L-L	9.81 ± 0.16	10.3 ± 0.12	10 ± 0.14	**0.050**	9.91 ± 0.15	10.28 ± 0.13	10.03 ± 0.13	**0.159**
	*p*-value	0.003	0.003	0.000		0.011	0.001	0.000	
No. of seeds per spikelet	H-H	3.04 ± 0.06	2.61 ± 0.08	2.57 ± 0.08	0.000	3.08 ± 0.06	2.65 ± 0.09	2.52 ± 0.06	0.000
	H-L	2.79 ± 0.06	2.55 ± 0.08	2.32 ± 0.08	0.001	2.77 ± 0.06	2.58 ± 0.08	2.29 ± 0.08	0.000
	L-H	2.67 ± 0.06	2.44 ± 0.08	2.2 ± 0.08	0.000	2.71 ± 0.07	2.43 ± 0.08	2.23 ± 0.08	0.000
	L-L	2.6 ± 0.07	2.37 ± 0.08	2.07 ± 0.08	0.000	2.63 ± 0.08	2.37 ± 0.08	2.1 ± 0.08	0.000
	*p*-value	0.000	**0.116**	0.000		0.000	**0.091**	0.003	
No. of seeds per plant	H-H	1298.916	1054.544	753.3955		1305.404	1059.576	795.438	
	H-L	1015.211	784.6605	563.7368		962.5196	753.8954	567.7368	
	L-H	821.025	515.7062	390.39		845.1406	564.7928	368.5354	
	L-L	644.0265	477.4365	362.25		645.0667	494.1095	389.6655	
Total no. of seeds from all plants	H-H	4221.477	5799.994	2448.535		3916.212	6092.562	2187.455	
	H-L	2538.028	3334.807	1268.408		2165.669	3392.529	1277.408	
	L-H	2052.563	1676.045	683.1825		1901.566	1976.775	644.9369	
	L-L	1288.053	1074.232	362.25		1290.133	1111.746	487.0819	

H-H, added fertilizer, watered regularly; H-L, added fertilizer, natural moisture; L-H, no fertilizer added, watered regularly; L-L, no fertilizer added, natural moisture; LS, spikelet type Left; RS, spikelet type Right; numbers 1, 2 or 3 following LS and RS indicate different seed groups. *p-*values in bold mean differences were not significant.

## Discussion

We found that seeds in the same position in Left and Right spikelets of *A. tauschii* had the same characteristics, thus they performed as mirror images between the two spikelet types. On the other hand, seed position in a spikelet had a significant effect on seed size, mass and germination; seedling emergence and survival; and plant size and seed production. These results support our first hypothesis that there is morphological and physiological variation between seeds in different positions in a spikelet. Since the natural germination unit is seeds that are inside the spikelet, we will first evaluate the effects of position of seeds while inside the spikelet on dormancy and germination. Then, we will consider the effects of seed position on seedling survival, plant size and seed production for seeds separated from the spikelets and sown in different environmental conditions. Finally, we will consider what the variation in seed characteristics may mean with regard to invasiveness of *A. tauschii*.

### Seed size and mass

Seed position in the spikelet of *A. tauschii* significantly affected seed size and mass. Among the three seeds in a spikelet, middle seeds (group 2) were significantly the longest and had maximum mass, while basal seeds (group 1) were significantly the shortest and had medium mass. In a spikelet, the basal and distal (group 3) seeds are on one side, and the middle seed is on the opposite side (Figure 1). Since the ovules above the middle seed usually do not develop into a seed, there is more space in the spikelet for growth/expansion of the middle than of the basal and distal seeds. The basal and distal seeds may have received fewer metabolites and/or growth regulators than the middle seeds due to competition between seeds for these resources, for example seeds of *A. kotschyi* (Wurzburger et al., 1976). Further, variation in seed size and mass related to seed position may be a function of variation in the timing and duration of reproductive development (Cheplick and Clay, 1989).

### Dormancy and germination

It is well known that seed size and mass can have effects on degree of dormancy, germination percentage and speed and growth of seedlings (see Baskin and Baskin, 2014). Thus, since seeds of *A. tauschii* vary in size, depending on position in the spikelet, we expected some differences in dormancy and germination. In the Poaceae, seeds differing in their position within a dispersal unit often vary in size and dormancy (Gosling et al., 1981; Dyer, 2004; Volis, 2016). In several Poaceae genera with a dispersal unit containing more than one seed, the first formed and therefore the better-developed (also larger) seed has lower dormancy than the later-developed seeds (Datta et al., 1970; Marañon, 1989; Dyer, 2004; Volis, 2016).

Some fresh seeds of *A. tauschii* enclosed by the spikelet germinated at each of the four temperature regimes in light and in dark, and the middle seeds germinated to the highest percentage in all conditions (Figures 5G–J). Regardless of test conditions, only a few or no basal seeds germinated, and the distal seeds only germinated to approximately ≤25% at 22/12°C in both light and dark. The ecological significance of these results is that when seeds mature in the field in early summer (June), temperatures in the habitat are above those required for germination, which would prevent even the middle seeds from germinating. During summer, however, seeds in the spikelets in the field afterripen, and by autumn the basal, middle and distal seeds enclosed by the spikelet can germinate over a range of temperatures in light and dark. Based on data for germination speed (index) of seeds removed from the spikelet after 12 months of storage, during which seeds afterripened, and incubated at the four temperature regimes (Figures 5K,L), we hypothesize that the order of seed germination speed in autumn is middle > distal > basal.

The hypothesized order of germination speed in autumn agrees with the amount of imbibition (i.e., increase in mass) and speed of imbibition of seeds: middle > distal > basal. Thus, middle seeds take up more water (and faster) and germinate faster than the basal and distal seeds, suggesting that they could germinate in early autumn in response to a precipitation event. Germination in early autumn could be followed by a rainless period, leading to death of the seedlings. If the seedlings for the middle seeds died, then with additional rains in autumn, it seems likely that the basal and distal seeds would germinate. Thus, differences in the basal, middle and distal seeds with regard to imbibition of water and germination speed after dormancy is broken potentially provide more than one opportunity for germination and seedling establishment in autumn.

From a broad perspective, the only kind of dormancy known to occur in the Poaceae is non-deep physiological dormancy (PD; Baskin and Baskin, 1998, 2001). In the present study, evidence for non-deep PD of seeds in the three positions of both spikelet types is 3-fold. (1) Most seeds in each position afterripened during dry storage under room conditions, resulting in high germination percentages over the range of temperatures in light and dark of seeds enclosed by the spikelets. (2) Seeds are water permeable. (3) Seeds of Poaceae have a fully developed embryo (Baskin and Baskin, 2021). Dormancy break of *A. tauschii* seeds *via* afterripening in dry storage during summer means seeds are capable of germinating in autumn if soil moisture and light/dark conditions are favorable, thus explaining the peak of germination in the field plots in autumn. Of course, autumn germination means that young plants of *A. tauschii* would be competing with those of wheat that also germinate in autumn. However, delaying germination until spring would result in young plants of *A. tauschii* competing with larger plants of wheat.

Dormancy break in summer and germination in autumn is a characteristic of seeds of winter annuals (Baskin and Baskin, 2001). During the afterripening period, germination percentages increased at all four temperature regimes tested, but temperature range for germination did not change. Thus, seeds have Type 6 of nondeep physiological dormancy (Nur et al., 2014; Soltani et al., 2017). If nondormant (afterripened) seeds of some winter annuals fail to germinate in autumn, they are induced into secondary dormancy by low temperatures during winter (Baskin and Baskin, 2014). It seems that at least some seeds of *A. tauschii* that fail to germinate in autumn are not induced into secondary dormancy because they germinated in spring. However, more research is needed to determine how many (and which) seeds within spikelets delay germination until spring.

### Seedling emergence and survival

**Figure 8:**
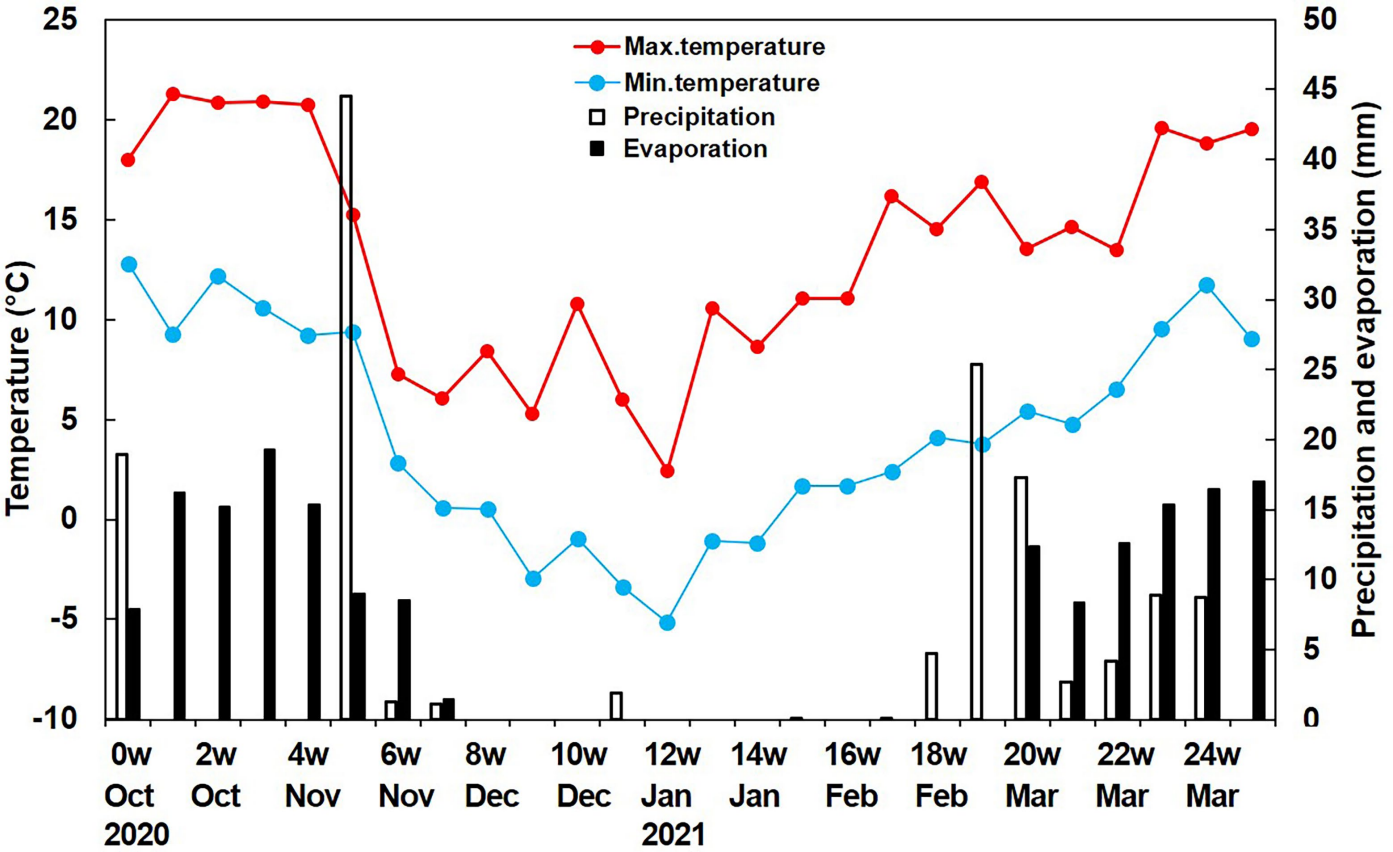
Weekly precipitation (rain and snow), evaporation and mean daily minimum and maximum temperatures during the study period.

Although seeds of *A. tauschii* can germinate in both autumn and spring, the peak of germination in our study was in autumn. By autumn, the temperatures in the habitat and those required for germination overlapped, and precipitation in autumn was more than 60 mm (Figure 8). Thus, seeds germinated in both the regularly watered and natural soil moisture conditions in our field experiment. However, water was more important than nutrients for seedling emergence, while nutrients were more important than water for seedling survival. The additional nutrients had a positive effect on seedling growth, resulting in high seedling survival of *A. tauschii.* Both seedling emergence percentages and seedling survival percentages of *A. tauschii* were the highest with both watering and fertilization; these conditions also had a highly synergistic effect on establishment of the invasive species *Centaurea solstitialis* and *A. triuncialis* (Eskelinen and Harrison, 2014). Thus, not surprisingly *A. tauschii* is a successful invasive species in wheat cropland in China under high moisture and nutrient-rich conditions.

Although, seedling emergence percentages in spring were significantly lower than those in autumn, seedling emergence percentages in both autumn and spring were best for the middle seeds and worst for the basal seeds. However, survival percentages for both autumn-and spring-emerging seedlings was best for the basal seeds and worst for the distal seeds. Some seeds in each position were capable of germinating in autumn, and plants survived until they matured, resulting in plants behaving as winter annuals. However, the delay of germination of some seeds of each of the three seed positions until spring indicates that the species can behave as a facultative winter annual (Baskin and Baskin, 1981, 2014).

### Plant size and seed production

For seeds from both spikelet types under the four experimental soil conditions, all growth indices were significantly higher for plants from seedlings emerging in autumn than for those emerging in spring. These differences may be the result of plants from autumn seedlings having a longer growth period than those from spring-germinated seedlings. For plants from autumn and spring seedlings from seeds of both spikelet types, all growth indices were significantly the highest under added fertilizer combined with regular watering (H-H) but lowest under no fertilizer combined with natural moisture condition (L-L), indicating that high levels of nutrients and water were very important for growth of *A. tauschii*, which is similar to the responses of the invasive species *A. triuncialis* (Eskelinen and Harrison, 2014). Although height of plants and number of spikelets per tiller of *A. tauschii* in all soil treatments were the highest for the middle seeds and the lowest for the basal seeds, number of tillers per plant and number of seeds per spikelet were the highest for the basal seeds and (except for a few cases) the lowest for the distal seeds. Thus, the surviving plants from the basal seeds produce a larger number of seeds per plant than those from either the middle or the distal seeds. However, seedling emergence percentages and total number of surviving plants from the middle seeds were higher than those from either basal or distal seeds, and all surviving plants from the basal and the distal seeds produced a lower total number of seeds than surviving plants from the middle seeds. Regardless of seed origin, timing of germination and soil conditions, each plant produced basal, middle and distal seeds, ensuring that there will be diversity in germination responses and in survival and reproductive strategies of offspring in the next generation.

In summary, there are considerable morphological and physiological differences between basal, middle and distal seeds in a spikelet of *A. tauschii*. These differences have the potential to spread germination over a period of time in autumn, depending on variation in soil moisture. Plants resulting from seeds in different positions in the spikelet vary with regard to survival, growth and seed production, and plants from seeds in different positions differ in their responses to soil fertility and moisture. We conclude that seed position in the spikelet likely makes a contribution to the invasiveness of *A. tauschii* because the variation between seeds in the different positions adds up to much flexibility in all stages of the life cycle—seed, seedling and growth/reproductive.

## Data availability statement

The raw data supporting the conclusions of this article will be made available by the authors, without undue reservation.

## Author contributions

AW and JD designed the experiments. AW performed the experiments and analyzed the data. AW, CB, JB, and JD wrote the manuscript. All authors reviewed the manuscript. All authors contributed to the article and approved the submitted version.

## Funding

This work was supported by the Major Public Welfare Projects in Henan Province (201300311300).

## Conflict of interest

The authors declare that the research was conducted in the absence of any commercial or financial relationships that could be construed as a potential conflict of interest.

## Publisher’s note

All claims expressed in this article are solely those of the authors and do not necessarily represent those of their affiliated organizations, or those of the publisher, the editors and the reviewers. Any product that may be evaluated in this article, or claim that may be made by its manufacturer, is not guaranteed or endorsed by the publisher.

## Footnotes

^1^http://www.cma.gov.cn (Accessed July 15, 2022).
